# Are variations in rates of attending cultural activities associated with population health in the United States?

**DOI:** 10.1186/1471-2458-7-226

**Published:** 2007-08-31

**Authors:** Anna V Wilkinson, Andrew J Waters, Lars Olov Bygren, Alvin R Tarlov

**Affiliations:** 1 Department of Epidemiology, Unit 1340, The University of Texas MD Anderson Cancer Center, 1155 Hermann Pressler Boulevard, Houston, TX 77030, USA; 2 Department of Behavioral Science, Unit 1330, The University of Texas MD Anderson Cancer Center, 1155 Hermann Pressler Boulevard, Houston, TX 77030 USA; 3 Department of Biosciences, Karolinska Institute, SE-171 77, Stockholm, Sweden; 4 Department of Community Health and Rehabilitation, University of Umeå, SE-901 87, Umeå, Sweden; 5 James A. Baker III Institute for Public Policy, Rice University, 6100 Main Street, Houston, TX 77005, USA; 6 Department of Medicine, University of Chicago, 540 North State Street, #3801, Chicago, Il 60610, USA

## Abstract

**Background:**

Population studies conducted in Sweden have revealed an association between attendance at cultural activities and health. Using data from US residents, we examined whether the association could be observed in the US.

**Methods:**

Participants in the current study included 1,244 individuals who participated in the 1998 General Social Survey.

**Results:**

A significant association between cultural activities and self-reported health (SRH) was observed, even after controlling for age, gender, marital status, race, number of children, subjective social class, employment status, household income, and educational attainment. Specifically, the more cultural activities people reported attending, the better was their SRH.

**Conclusion:**

The data confirm that an association between cultural activity and health is present in a US sample. The data do not mean that the association is causal, but they suggest that further longitudinal research is warranted.

## Background

Research conducted in Sweden has explored the relationship between attending cultural activities and both mortality and self-rated health (SRH). The Swedish studies suggest that there are health benefits to be gained by participating in different types of cultural activities. Bygren et al. [1] reported that people who frequentltended cultural activities during a nine-year period had better survival odds than those who rarely attended. Based on the same cohort, but after a 14-year time lapse, the researchers found a higher mortality risk for people who rarely went to the cinema, concerts, museums or art exhibitions compared with those who went frequently [2]. Both analyses controlled for gender, age, education, disposable income, social contacts, presence of a long-term disease, smoking and exercise patterns. In a third analysis the researchers [3] constructed a cultural attendance indicator that reflected attendance and frequency of attendance at cinemas, theatres, concerts and live music, museums and art galleries, which they used to examine changes in self-reported health associated with changes in attendance of cultural activities over an eight-year period. People who became less active over the eight-year period or who were inactive at both time periods were more likely to report being in poor health compared to people who were active at both time periods or who became more active over the eight years. This analysis controlled for gender, education, home ownership, urbanity, reading, making music, and health status at the first time period.

A Canadian study of police and emergency response workers supported these findings. The more frequently participants attended cultural activities during their leisure time, the better their physical health [4]. Research in the US has hitherto focused on the relationships between health and leisure time physical activity [5,6], volunteering [7,8], and religious attendance [9,10]. To the best of our knowledge, no previous study has examined the association between cultural activity and health in the US population. Given that 1) there are striking differences in social structure and health outcomes between Sweden and the United States [11,12]; 2) there are few characteristics that are as strongly associated with SES as attendance at cultural activities [13]; and 3) the well documented relationship between health and socio-economic status (SES) is not fully explained by differences in access to health care [14], work conditions [15], social ties [16], and health behaviors [17], we examined whether the association between cultural activity and self-reported health could be observed in the US population.

## Methods

The analysis utilizes public use data from the 1998 General Social Survey (GSS). The GSS is an annual survey of attitudes toward social issues that began in 1972 and comprises a core set of demographic and attitudinal questions as well as rotating topical modules [18]. It is a national area probability sample of non-institutionalized adults residing in the United Status. Data are collected by in-person interviews and verbal informed consent was obtained from all study participants. In 1998 a random sample of 1,435 individuals, 18–89 years old, completed a topical module reporting on the types of cultural activities attended in the previous year. The overall response rate was 75.6%. All aspects of this study received approval from the Institutional Review Board at the University of Chicago.

### Outcome variable: Self-Rated Health (SRH)

Participants answered the question "In general how would you rate your health?" Responses were made on a four-point scale: excellent, good, fair, or poor. Consistent with the Swedish study [3], we combined excellent/good into one category (coded as 1) and fair/poor into a second category (coded as 0).

### Culture attendance

Participants were asked "Next I'd like to ask about some leisure or recreational activities that people do during their free time. As I read each activity, can you tell me if it is something you have done in the past twelve months..." attended 1) art exhibits (37.9%), 2) dance performances (20.4%), 3) operas or classical recitals (17.1%), 4) movies (67.5%), 5) live popular music (39.2%), or 6) plays (theatre) (24.1%) (% endorsements). We also created a variable (Cultural Activity) that reflected cumulative cultural activity during the year by summing the Yes responses. A person who reported attending none of the cultural activities received a score of 0, while somebody who reported attending all of the six activities received a score of 6. Cultural Activity was entered as a continuous variable in analyses (Mean = 2.06, SD = 1.67).

### Control variables

Cultural preferences and activities are influenced by many factors including social class, gender, race, education, and age [13]. SRH also varies with gender, age, marital status, race, employment status and SES [6,19]. We therefore controlled for age, gender, marital status, race, number of children, subjective social class, employment status, household income and years of education. Age (range 18 – 89), number of children (range 0 – 8+), household income (range $1,000 – $120,000+), and years of education (range 0 – 20) were entered as continuous variables. Gender (female, male), marital status (never married, divorced or separated, widowed, married), race (black, other, white), subjective social class (lower class, working class, middle class, upper class), and employment status (not currently working, retired, homemaker, employed), were entered as class variables (reference category is listed last).

### Statistical methods

We used univariable logistic regression (proc logistic in SAS) to examine the relationships between each control variable/Cultural Activity and SRH. We used the general linear model (proc glm in SAS) to examine the relationships between control variables and Cultural Activity; for each control variable we report the unstandardized parameter estimate (b).

Multivariable logistic regression analyses were used to establish if there was an association between attending cultural activities and SRH. Models were built for a) each type of activity separately and b) Cultural Activity. The first set of models (I) was adjusted for the demographic characteristics including age, gender, martial status, race, and number of children. The second set of models (II) further adjusted for aspects of SES including subjective social class, employment status, household income, and years of education. In all models, all covariates were entered simultaneously. All statistical tests were 2-tailed and all analyses were conducted in SAS [20].

### Sample size

Of the 1,435 respondents who completed the topical module, 21 individuals were excluded because of missing data on SRH, age, subjective social class, and number of children. Another 170 individuals were excluded because of missing data on income and education. Thus, the final sample size for analysis was 1,244 (87% of respondents).

## Results

### Associations with SRH and Cultural Activity

Summary statistics are presented in Table 1. Univariable logistic regression models revealed that age (Odds Ratio (OR): 0.97, 95% Confidence Interval (CI): 0.96 – 0.98, p < 0.01), marital status (p < 0.01), subjective social class (p < 0.01), employment status (p < 0.01), income level (OR: 1.12, CI: 1.09 – 1.14, p < 0.01), years of education (OR: 1.22, CI: 1.16 – 1.29, P < 0.01), and Cultural Activity (OR: 1.41, CI: 1.28 – 1.55, p < 0.01) all significantly predicted SRH. Being widowed (OR: 0.32, CI: 0.20 – 0.49) or divorced (OR: 0.58, CI: 0.40 – 0.83) was associated with lower SRH (vs. being married). Self-identifying as lower class was associated with lower SRH (vs. self-identifying as upper class) (OR: 0.14, CI: 0.05 – 0.35). Being a homemaker (OR: 0.32, CI: 0.21 – 0.48), being retired (OR: 0.25, CI: 0.17 – 0.36), or not working (OR: 0.32, CI: 0.20 – 0.51) was associated with lower SRH (vs. being employed).

**Table 1:** Self-Rated health and cultural activity by demographic characteristics (N = 1,244)

**Characteristic**	**SRH**		**No. of Cultural Activities Attended**			
		
	**N**	**%**	**0**	**1 – 2**	**3 – 4**	**5 – 6**
**Age**						
18–24	104	85.6	5.8	52.9	31.7	9.6
25–34	294	87.1	12.9	51.0	26.9	9.2
35–44	296	83.8	14.9	46.3	27.4	11.5
45–54	221	77.4	18.1	43.4	24.9	13.6
55–64	138	76.1	29.7	34.1	20.3	15.9
65–74	116	66.4	35.3	38.8	15.5	10.3
75+	75	53.3	56.0	32.0	8.0	4.0
**Gender**						
Male	570	78.8	20.0	47.7	21.4	10.9
Female	674	79.7	20.5	41.8	26.4	11.3
**Marital Status**						
Married	586	82.9	20.6	44.0	22.9	12.5
Widowed	109	60.6	43.1	38.5	15.6	2.8
Div/Sep	232	73.7	17.6	51.7	22.8	7.7
Never married	317	83.0	13.6	42.3	30.3	13.9
**Race**						
White	984	80.3	18.5	43.7	25.9	11.9
Black	174	76.4	29.9	50.0	14.9	5.2
Other	86	73.3	20.9	43.0	22.1	14.0
**No. of Children**						
None	271	79.2	--	--	--	--
One	176	82.2	--	--	--	--
Two or more	438	78.3	--	--	--	--
Mean (SD)	1.8 (1.7)		1.7 (1.4)	1.9 (1.8)	1.7 (1.6)	1.9 (1.7)
Range	0 to 8			0 to 8		
**Subjective Social Class**						
Lower	73	43.8	37.0	42.5	16.4	4.1
Working	564	78.0	22.0	51.8	19.9	6.4
Middle	560	84.6	16.6	38.9	28.9	15.5
Upper	47	85.1	17.0	27.7	29.8	25.5
**Employment Status**						
Employed	849	86.2	14.8	45.1	26.9	13.2
Not working	99	66.7	24.2	45.5	22.2	8.1
Retired	161	60.9	37.9	38.5	16.1	7.5
Homemaker	135	66.7	30.4	47.4	17.8	4.4
**HH Income ($)**						
< 14,999	257	63.0	39.7	38.9	15.2	6.2
15,000 – 29,999	314	76.1	19.4	48.4	24.5	7.6
30,000–49,999	313	83.4	18.5	47.0	25.2	9.3
50,000–74,999	182	89.6	9.9	54.4	22.5	13.2
≥ 75,000	178	90.4	7.3	31.5	36.0	25.3
**Education**						
< High school	209	60.3	44.5	45.9	9.1	--
High school	352	73.6	28.7	53.1	16.2	2.0
Some college	360	86.4	10.8	49.7	27.8	11.7
College degree	323	89.8	5.9	28.5	38.4	27.2
**No. Cultural Activities**						
0 events	252	64.7	--	--	--	--
1 event	295	75.3	--	--	--	--
2 events	259	84.2	--	--	--	--
3 events	180	83.3	--	--	--	--
4 events	120	90.8	--	--	--	--
5 events	98	89.8	--	--	--	--
6 events	40	90.0	--	--	--	--

Data shown are percentages reporting good/excellent SRH (left side), and percentages reported 0, 1–2, 3–4, 5–6 cultural activities (right side).

Gender (b = 0.25, Standard Error (SE) = 0.09, p < 0.01), marital status (p < 0.01), race (p < 0.05), subjective social class (p < 0.01), income (b = 0.034, SE = 0.01, p < 0.01), and years of education (b = 0.22, SE = 0.016, p < 0.01) were independently associated with Cultural Activity (in a multivariable model). The never-married had higher cultural activity scores than the married (b = 0.40, SE = 0.12, p < 0.01). Blacks had lower cultural activity scores than whites (b = -0.33, SE = 0.12, p < 0.01). Individuals self-identifying as lower class (b = -0.58, SE = 0.28, p < 0.05), or as working class (b = -0.77, SE = 0.22, p < 0.01) had lower cultural activity scores than those self-identifying as upper class.

### Multivariable logistic regression models

In the first set of models, attendance at all of the individual events, except the opera or classical music recital, was significantly associated with SRH (Table 2, left side). Cultural Activity was also significantly associated with SRH. In the second set of models, which were further adjusted for subjective social class, employment status, household income, and years of education, only Cultural Activity maintained significance (Table 2, right side). Each additional event attended was associated with a 12% (OR: 1.12, CI: 1.01 – 1.26) increased chance of reporting good/excellent health. Several control variables, i.e., age (p < 0.01), gender (p < 0.05), subjective social class (p < 0.01), employment status (p < 0.01), and household income (p < 0.05), were also significant independent predictors of SRH. Specifically, being younger, being female, self-identifying as upper class (vs. lower class), being employed, and reporting a higher household income all significantly predicted better SRH (Table 3).

**Table 2:** Relationships between cultural activities and self-reported health (N = 1,244)

**Type of event**	**Model I**^**1**^	**Model II**^**2**^
	OR (95% CI)	OR (95% CI)
Art Exhibit	1.82 (1.32–2.50)	1.25 (0.88–1.77)
Dance Performance	2.15 (1.41–3.27)	1.44 (0.92–2.26)
Opera or Classical Recital	1.46 (0.97–2.20)	0.88 (0.56–1.39)
Movie	1.84 (1.35–2.51)	1.16 (0.83–1.64)
Live Music	1.78 (1.29–2.47)	1.36 (0.96–1.92)
Theatre	2.21 (1.49–3.27)	1.44 (0.94–2.20)
Cultural Activity	1.31 (1.19–1.45)	1.12 (1.01–1.26)

Data shown are adjusted Odds Ratios (95% Confidence Intervals) from logistic regression models relating Cultural Activity to SRH (1 = good/excellent; 0 = fair/poor). Each event type is tested in a separate model. Cultural Activity is also tested in a separate model.

^1^Model I models are adjusted for age, gender, martial status, race, and number of children.

^2^Model II models are further adjusted for subjective social class, employment status, household income, and education.

**Table 3:** Predictors of self-reported health (N = 1,244).

**Characteristic**	**Odds Ratio**	**95% Confidence Interval**	**P-Value**
**Age**			
Per year	0.97	0.96 – 0.99	< 0.01
**Gender**			
Male	1.00 (reference)		
Female	1.42	1.02 – 1.98	< 0.05
**Marital Status**			
Married	1.00 (reference)		
Widowed	0.97	0.55 – 1.71	0.91
Div/Sep	0.71	0.46 – 1.09	0.12
Never married	0.77	0.48 – 1.24	0.29
**Race**			
White	1.00 (reference)		
Black	1.18	0.76 – 1.85	0.46
Other	0.60	0.34 – 1.04	0.07
**No. of Children**			
Per child	0.99	0.90 – 1.08	0.79
**Subjective Social Class**			
Lower	0.25	0.09 – 0.69	< 0.01
Working	0.59	0.24 – 1.47	0.26
Middle	0.92	0.37 – 2.29	0.86
Upper	1.00 (reference)		
**Employment Status**			
Employed	1.00 (reference)		
Not working	0.50	0.30 – 0.84	< 0.01
Retired	0.56	0.33 – 0.95	< 0.05
Homemaker	0.48	0.29 – 0.79	< 0.01
**HH Income ($)**			
Per $1000	1.04	1.01 – 1.08	< 0.05
**Education**			
Per year	1.06	1.00 – 1.13	0.05
**No. Cultural Activities**			
Per activity	1.12	1.01 – 1.26	< 0.05

Data shown are adjusted Odds Ratio (95% Confidence Intervals) from a multivariable logistic regression model relating Cultural Activity to SRH (1 = good/excellent; 0 = fair/poor).

## Discussion

The more cultural activities people reported attending, the better was their SRH. This remained true after controlling for several potentially confounding variables such as age, gender, martial status, race, number of children, subjective social class, employment status, household income, and years of education. Confidence in the findings is increased by the fact that similar results have been observed in a Swedish study [3].

Studies investigating social activities have repeatedly demonstrated that such activities have health benefits. Frequently these health benefits are assumed to derive from increased activity levels that result in improvements in cardiovascular functioning [21,22]. However, some researchers report that the health benefits gained from social activities that do not directly improve physical fitness may be as great as those gained from physical activities that directly increase fitness [23]. Although this study did not directly assess mechanisms linking attending cultural activities with health, it seems plausible that the benefits derived from attending cultural activities are related, partly at least, to social aspects of the activities. People frequently attend cultural events with friends; being part of a social group that provides social, emotional and instrumental support has positive health benefits [24]. That said, cultural stimulation likely has an effect on health in its own right. Results from an animal experiment demonstrate that environmental stimulation from social interactions has a different effect on the brain than that from inanimate aspects of the environment [25]. This could be analogous to the stimulation from viewing art in the company of friends, and speaks to potential pathways through which cultural activities may influence health.

Indeed, the arts have been used for several decades as a therapeutic health-enhancing tool for individuals with no reports of adverse effects on the health and well-being of the participants. Studies that have evaluated the potential of the arts as a therapeutic health-enhancing tool have demonstrated that music, art, and mental imagery can have a beneficial impact on both mental and physical health [26-29]. For example, results from a case-control intervention with elderly women found that compared to the controls, women who had received an art intervention reported improvements on several self-reported indicators of health status and decreased systolic blood pressure [28].

It is possible that the reported health benefits associated with art therapies are related to reduced levels of stress. Stress reduction decreases oxidative DNA-damage and the formation of 8-hydroxydeoxyguanosine, elevated levels of which are linked to the development of disease, including cancer [30]. Participating in leisure time activities is an effective mechanism of coping with stress and engaging in activities that are perceived to be meaningful may be particularly important during periods of stress [4]. Therefore it also is possible that attending cultural activities serves as a buffer against harmful stress, thereby lowering disease risk.

The study had a number of limitations. First, the data are cross-sectional, meaning that the direction of causality is uncertain. It is possible that good health increases motivation and the ability to attend cultural activities. Second, the data were exclusively based on self-report. It would be helpful if future studies reported a clinical health endpoint as well as SRH. Third, the cultural activities examined were limited to those assessed in the GSS. This may have resulted in the misclassification of people who attended cultural activities that were not assessed by the GSS. In future research it will be helpful to use a broader range of cultural events (e.g. visiting museums, zoos, and aquariums, as well as attending ethnic festivals and sporting events). Fourth, information was not available on how frequently participants attended each event. The Swedish studies included an assessment of intensity and demonstrated that changes in the intensity of cultural consumption were associated with changes in health status [3]. Fifth, none of the fully-adjusted models that investigated each type of event separately demonstrated a significant relationship between any one event and SRH. The results therefore are silent as to whether attending each type of event is particularly strongly associated with better health. Sixth, the analysis did not include several important confounding variables, such as exercise patterns and dietary behaviors which influence SRH [31], and the presence of serious illness [32], which influences SRH and could also influence attendance at cultural activities. Last, the Cultural Activity effect was attenuated when more confounders were included in the models (Table 2). We do not know what residual confounding remains.

## Conclusion

In conclusion, despite these limitations, our results suggest that further research is warranted on the relationship between cultural activities and health. Such research should use longitudinal experimental methods and clinical end-points. If such future research suggests that participating in cultural activities does have health benefits, this may have implications for socio-cultural policies designed to improve health.

## Competing interests

The author(s) declare that they have no competing interests.

## Authors' contributions

AVW & AJW completed the analyses and interpreted the results. LOB interpreted the results and provided critical revisions. ART conceived of the study and provided critical revisions.

## Pre-publication history

The pre-publication history for this paper can be accessed here:


